# Identification by PCR-FRLP of bacteria present in the colostrum of women residing in Barranquilla, Colombia

**DOI:** 10.17843/rpmesp.2025.422.14321

**Published:** 2025-06-11

**Authors:** Luz A. Sarmiento-Rubiano, Leidys Goenaga, Marianella Suarez-Marenco, Clara Gutierrez-Castañeda, Carmen M. Sarmiento, Jimmy Becerra Enríquez

**Affiliations:** 1 Food and Human Behavior Research Group, Metropolitan University, Barranquilla, Colombia. Metropolitan University, Barranquilla Food and Human Behavior Research Group Metropolitan University Barranquilla Colombia; 2 Ecological and Agro-industrial Management Research Group, Microbiology Program, Free University, Barranquilla, Colombia. Free University Ecological and Agro-industrial Management Research Group Microbiology Program Free University Barranquilla Colombia; 3 Preventive and Community Medicine, University of Sinú, Cartagena, Colombia. University of Sinú Preventive and Community Medicine University of Sinú Cartagena Colombia

**Keywords:** Colostrum, Polymorphism, Restriction Fragment Length, Microbiota, Breast Feeding

## Abstract

With the aim of isolating and identifying bacteria present in the colostrum of women in the city of Barranquilla, located in the Colombian Caribbean, we carried out a descriptive study on 55 colostrum samples, which were cultured on M17, MRS, and TOS agar and incubated under aerobic and anaerobic conditions. A total of 350 microorganisms were isolated, of which 296 were identified at the genus level by PCR-RFLP with the enzymes HaeIII and RsaI. The online program kodebio.shinyapps.io/RFLP-inator was used for the in-silico identification of the isolates. Seven hypothetical bacterial genera were identified: *Staphylococcus, Lactobacillus, Enterococcus, Bifidobacterium, Corynobacterium, Streptococcus*, and *Leuconostoc*, with *Staphylococcus, Lactobacillus, and Enterococcus* being the most dominant, representing 61.1% of the identified microorganisms. Our results are a starting point for understanding the microbial composition of human colostrum, considering the particular context and environmental conditions of the Colombian Caribbean.

## INTRODUCTION

Currently, more than 590 bacterial genera have been identified in human milk, belonging mainly to the *phyla Firmicutes, Actinobacteria*, and *Protebacteria*, with the genera *Staphylococcus, Streptococcus, Lactobacillus, Pseudomonas, Bifidobacterium*, *Corynebacterium, Enterococcus, Acinetobacter*, and *Clostridium*, among others, being particularly dominant ^1^.

Human milk has all the components and nutrients necessary for the proper growth, development, and protection of newborns, and is their main source of commensal, mutualistic, and potentially probiotic bacteria ^2^. The bacteria provided by breast milk perform various functions, including: reducing the incidence and severity of infections ^3^; maturing the immune system ^4^; defending against pathogenic bacteria through the presence of bacterial phages ^5^; and exposure to the maternal milk microbiome promoting the intestinal health of the newborn and their physical and mental development ^6^.

The composition of the human milk microbiota varies according to the stage of lactation and factors such as the mother’s hygiene and diet, her gut microbiota, antibiotic use, geographical location, lifestyle, and genetic, epigenetic, and ethnic factors ^7^. Colostrum is produced by the mammary glands from the moment of birth and for six days thereafter. It is rich in cytokines, antimicrobial peptides, antibodies, hormones, and various bioactive compounds ^2^. The colostrum microbiota is strongly associated with the maternal environment, as well as the type of delivery (natural or cesarean), and the evolution from colostrum to mature milk involves significant changes in its microbiota, in which the interaction of the infant’s emerging oral microbiome with the mammary gland is fundamental ^8^.

Studying microorganisms in human milk under different environmental and population conditions contributes to our knowledge of this fluid and its potential impact on the health of infants and mothers. Culture-dependent and culture-independent techniques allow the identification of a wide variety of bacteria, each with advantages and disadvantages that are evident in the obtained results. However, culture techniques allow the isolation and preservation of microorganisms with potential probiotic characteristics. This study aimed to isolate and identify bacteria from the colostrum of women in the city of Barranquilla, in the Colombian Caribbean, using the culture technique and subsequent identification by PCR-FRLP (Restriction Fragment Length Polymorphism).

**KEY MESSAGES**

Motivation for the study. There is little information on the microbiota found in the colostrum of women living in the Colombian Caribbean. Identifying and isolating microorganisms in this fluid is of interest to both medicine and industry.

Main findings. The dominant bacterial genera found in colostrum were *Staphylococcus, Lactobacillus,* and *Enterococcus*. Microorganisms with probiotic potential were isolated and should be studied in greater detail.

Public health implications. Understanding the microbiota found in colostrum allows us to understand its contribution to infant health and develop strategies to enhance the beneficial impact of these microorganisms on their development.

## THE STUDY

A descriptive cross-sectional study was conducted, including 55 postpartum women who were treated at the maternity ward of the Metropolitan Hospital in the city of Barranquilla (Colombian Caribbean) and who met the following inclusion criteria: being of legal age, having had a minimum of 37 weeks of gestation, vaginal delivery without complications or antibiotic use, being in good health, and having voluntarily agreed to participate by donating 3 to 5 ml of colostrum, collected between 12 and 48 hours postpartum. The colostrum was obtained after washing the nipples with mild soap and sterile water, using an electric breast pump (Medela Swing) under sterile conditions. The first two drops of colostrum were discarded, and 3 to 5 ml of sample were collected in a sterile container and stored at -80°C until analysis.

To isolate bacteria from colostrum, 100 µl of sample was directly seeded onto MRS (Man Rogosa Sharpe, for the isolation of acid-lactic bacteria), M17 (for lactic *Streptococcus*) and Propionate TOS agar (for bifidobacteria) culture media. The agar plates were incubated at 37 °C for 72 hours (MRS and M17 under aerobic and anaerobic conditions—Oxoid Anaerogen system and TOS under anaerobic conditions only). The colonies isolated from the different cultures were morphologically identified by Gram staining, re-cultured in liquid culture medium according to the agar of origin or in Brain Heart Infusion (BHI) broth, and cryopreserved at -80 °C with 20% glycerol for future studies.

The gender identification of bacteria isolated from colostrum was performed using PCR-RFLP. Approximately 1508 base pairs of r16S DNA were amplified by colony PCR with universal primers 27F: 5'-AGAGTTTGATCCTGGCTCAG-3' and 1492R: 5'-GGTTACCTTGTTACGACTT-3' ^9^. Each 50 µl PCR reaction contained: 1X buffer (BIOTOOLS), 1.5 mM MgCl2, 200 μM dNTPs, 20 pmol of each primer, 1U Taq polymerase (BIOTOOLS), and a small portion of colony added with a sterile toothpick. *Lactococcus lactis* NZ900 was used as a positive control for the reaction. The amplification program was as follows: five minutes at 96 °C; 35 cycles: 95 °C for 30 seconds, 60 °C for 30 seconds, 72 °C for one minute; and one cycle of 72 °C for seven minutes (BIO-RAD Thermocycler). The PCR products were visualized on 0.8% agarose gels stained with Syber-Green. Electrophoresis was performed with 1X TAE buffer at 80V for 30 minutes (Bio-Rad horizontal electrophoresis).

For RFLP, PCR amplificates were digested in separate reactions with the enzymes HaeIII (GC^GC) and RsaI (GT^AC) according to the manufacturer’s recommendations (New England Biolab) (10 µl of PCR product, 2 units of enzyme, 2 µl 10X buffer in a final volume of 20 µl). The reactions were incubated for 3 hours at 37 °C. The digestion products were subjected to electrophoresis in 2% agarose gels with Syber-Green and the generated band profile was visualized on a Biorrad ChemiDoc™ XRS photodocumenter. The band sizes obtained were compared with the HyperLadder™ 100pb DNA-Bioline molecular weight marker.

The resulting band profiles were classified into eight groups of microorganisms that showed identical restriction profiles with the two enzymes. Using the online program kodebio.shinyapps.io/RFLP-inator, which allows bacterial identification based on *in silico* simulation of 16S DNA restriction profiles, it was possible to identify the isolates obtained at the genus level. *Lactococcus lactis* subsp. cremoris NZ9000 (GenBank NCBI sequence NC_017949.1) was used as a positive control, whose restriction profiles with HaeIII and RsaI were verified *in silico*.

We conducted a descriptive statistical analysis. Microbial growth is expressed as log^10^ CFU/ml. The identification of microorganisms by PCR-RFLP is reported as relative frequencies.

The research protocol for this study was approved by the Ethics Committee of the Metropolitan University (code 0340517) and all participants signed an informed consent form in accordance with Resolution 8430 of 1993 of the Colombian Ministry of Health and Social Protection and the Declaration of Helsinki.

## FINDINGS

Of the 55 colostrum samples cultured, only 90.9% (n=50) showed microbial growth in any of the culture media under the described conditions. The average number of microorganisms isolated from each colostrum was 2.73 log^10^ CFU/ml (range 1.30 to 3.47). Microbial growth in each of the culture media and evaluated conditions had an average value of 2.39 log^10^ CFU/ml (Table 1). Taking into account the different culture conditions and differential morphology of the colonies, it was possible to isolate 350 microorganisms. Gram staining was used to determine the microscopic morphology of the isolates; in M17 medium, mainly Gram-positive cocci were observed, in MRS medium, Gram-positive coccobacilli and Gram-positive bacilli, and in TOS medium, Gram-positive cocci and coccobacilli.

**Table 1:** Number of microorganisms isolated from colostrum samples from each culture medium and different conditions.

Culture medium	Condition	Samples with growth n=50 (%)	Average log^10^ CFU /ml	Number of isolated microorganisms (n=350)
M17	Aerobic	40 (80)	2.42	128
	Anaerobic	28 (56)	2.27	72
MRS	Aerobic	16 (32)	2.41	53
	Anaerobic	8 (16)	2.59	27
TOS	Anaerobic	23 (46)	2.36	70

CFU: colony-forming units, M17: M17 agar culture medium, MRS: Man Rogosa Sharpe medium, TOS: propionate agar medium.

**Figure 1:**
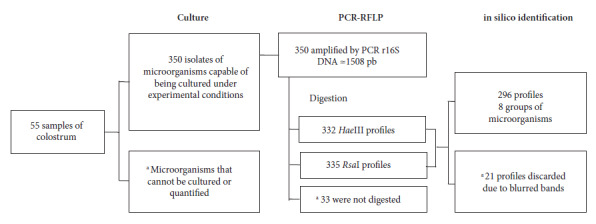
Flowchart of the process of culturing and identifying microorganisms in human colostrum.

^a^ Shows the causes of exclusion of microorganisms in the process.

PCR amplification (≈1508 bp) of r16S DNA was possible in all the isolated microorganisms. Digestion with the enzymes *HaeIII* and *RsaI* resulted in 332 and 335 band profiles. Since not all isolates showed a defined pattern (diffuse bands, imperceptible bands, or non-digestion of the DNA fragment), only 296 isolates were selected for identification (Figure 1). Since many of the bacteria presented the same band profile with both enzymes, the profiles were grouped, obtaining eight restriction patterns from the 296 microorganisms (Table 2).

**Table 2:** Identification of bacterial genera isolated from colostrum samples using PCR-RFLP.

Pattern	Culture media	Gram stain	Identification of band profiles by RFLP-inator			% MI (n=296)
			^a^ ***Hae*III**	^a^ ***Rsa*I**	Hypothetical microorganisms	
1	M17 aerobiosis M17 anaerobiosis	Gram + Cocci	1200, 310	630, 490, 380	*Hae*III: S004065075 *Staphylococcus aureus* 08BA02176 *Rsa*I: S000404448 uncultured rumen bacterium BE38	16.4
2	M17 aerobiosis M17 anaerobiosis	Gram + Cocci	1200, 310	500, 490, 410, 120	*Hae*III: S004443882 *Staphylococcus epidermidis* SEI *Rsa*I: S001095749 *Staphylococcus epidermidis* SSL08	20.2
3	M17 anaerobiosis M17 anaerobiosis	Gram + Cocci	600, 460, 290, 120, 30	900, 350, 150, 120	*Hae*III: S000892225 *Enterococcus mundtii* HDYM-22 *Rsa*I: S002949963 *Enterococcus* sp. PPC34A	12.4
4	M17 anaerobiosis	Gram + Cocci	580 450, 310, 120	620, 360, 260, 150, 120	*Hae*III: S000470127 *Streptococcus salivarius* subsp. null DL1 *Rsa*I: S002032101 *Streptococcus* sp.	8.2
5	MRS aerobiosis MRS anaerobiosis	Gram + bacilli	570, 460, 330, 120, 30	900, 350, 140, 110	*Hae*III: S003260210 *Lactobacillus pentosus* ZU 28 *Rsa*I: S003260206 *Lactobacillus pentosu*s ZU 24	15.1
6	MRS anaerobiosis	Gram + Cocci-bacilli	1100, 270, 70, 45	490, 400, 120, 100	*Hae*III: S000430949 *Leuconostoc pseudomesenteroides* RO1 *Rsa*I: S000978801 *Leuconostoc pseudomesenteroides* QL28	7.7
7	TOS anaerobiosis	Gram + bacilli	460, 360, 160, 95, 85, 65, 55, 35	410-210-160-150-115-85	*Hae*III: S001576581 *Corynebacterium* sp. ICIRC105 *Rsa*I: S000721364 *Corynebacterium aurimucosu*m AE1-3	8.5
8	TOS anaerobiosis	Gram + bacilli	260, 200, 150, 100, 90, 70, 50, 30, 20	630, 360, 250, 150, 120	*Hae*III: S000871434 *Bifidobacterium animalis* subsp. lactis IDCC 4301 *Rsa*I: S000871434 *Bifidobacterium animalis* subsp. lactis IDCC 4301	11.5
Control	M17 anaerobiosis	Gram + Diplococci	457, 410, 309, 187, 123, 22	890, 355, 146, 83, 34	*Lactococcus lactis* subsp. cremoris NZ9000, (NC_017949.1)	

The band profiles obtained with each digestion enzyme are shown according to the band sizes generated.

a HaeIII and RsaI are restriction enzymes used in digestion.

MI: identified microorganisms. RFLP: Restriction Fragment Length Polymorphism, M17: Agar M17 culture medium, MRS: Man Rogosa Sharpe medium, TOS: propionate agar medium.

Bacterial genera were identified based on their growth in the culture medium, Gram staining morphology, and the results of *in silico* band pattern analysis using the program kodebio.shinyapps.io/RFLP-inator.

The highest number of microorganisms (57.2%, n=200) was isolated and identified from the M17 culture medium. We identified bacteria of the genus *Staphylococcus* (patterns 1 and 2) growing under aerobic and anaerobic conditions, which showed a band profile common to digestion with *HaeIII*, but different with *RsaI*; and bacteria of the genus *Enterococcus* (pattern 3). Microorganisms grown on M17 agar only under anaerobic conditions accounted for 8.2% of the isolates, which were identified as bacteria of the genus *Streptococcus* (pattern 4).

We found that 22.8% of the identified microorganisms were isolated in the MRS medium, under aerobic and anaerobic conditions. *Lactobacillus* (pattern 5) was identified, as well as *Leuconostoc* (pattern 6), which grew only under anaerobic conditions. The bacteria recovered and identified in the TOS medium under anaerobic conditions (band patterns 7 and 8), representing 20% of the isolates, belonged to the genera *Corynebacterium* sp. and *Bifidobacterium*, respectively (Table 2).

## DISCUSSION

In this study, seven hypothetical bacterial genera were isolated and identified by PCR-RFLP from 50 human colostrum samples, belonging to the genera *Staphylococcus*, *Lactobacillus*, *Enterococcus, Bifidobacterium, Corynobacterium*, *Streptococcus*, and *Leuconostoc*, with *Staphylococcus, Lactobacillus* and *Enterococcus*, corresponding to 61.1% of the 296 microorganisms identified from 350 isolates.

Other authors, who used culture-dependent methods, have identified *Staphylococcus, Streptococcus, Corynobacterium*, and *Cutibacterium* as the dominant bacterial genera in human milk, and in smaller proportions *Lactococcus*, *Enterococcus*, *Lactobacillus*, *Leuconostoc*, *Weisella*, and *Bifidobacterium*^2^^,^^10^. As in this study, *Staphylococcus*-predominant milk flora has been previously reported. For example, in women from urban areas of China, r16S DNA sequencing showed that more than 40% of the milk microbiota corresponded to bacteria of the genera *Staphylococcus* and *Streptococcus*^11^. A meta-analysis that included twelve studies from different parts of the world found that the dominant genera in milk, regardless of geographical location, were *Staphylococcus* and *Streptococcus*^12^. In this regard, it should be noted that the microbiota evolves from colostrum to mature milk and that studies such as that by Jiménez *et al*. in 2008, in colostrum from 36 Spanish women, found *Staphylococcus* and *Enterococcus* to be the dominant genera, with a lower proportion of *Lactobacillus*^13^. These results are consistent with those found in this study, in which *Enterococcus* and *Lactobacillus* were the most frequent genera after *Staphylococcus*.

The mother’s diet, diabetes, obesity, and even the sex of the baby, among other factors, have been identified as influencing the composition of the colostrum microbiota ^14^. Xie *et al*., evaluating 97 healthy mothers on the Chinese island of Hainan, found that the ethnic origin of individuals may be an important factor associated with the diversity of the colostrum microbiome ^15^. In Colombia, and specifically in the Caribbean, there is great ethnic and cultural diversity characterized by a mixture of African, European, and Native American ancestry ^16^. This diversity gives rise to such varied ethnic and genetic profiles that it would be very difficult to establish relationships between the milk microbiota and ethnicity, especially when only gender identification of the species found in colostrum is available. With regard to maternal diet, it is known that the consumption of macronutrients, mainly fat, modifies the nutritional quality of colostrum ^17^. The availability of nutrients in both the milk fluid and the infant’s intestinal mucosa affects the variety and quantity of existing bacterial populations. In this study, maternal diet was not evaluated. In Colombia, there are few studies on human milk microbiota. Among them, Londoño-Sierra *et al*., evaluating 101 lactating women in the city of Medellín, demonstrated a direct relationship between nutritional status and maternal diet with the dominant microbial genera in milk ^18^.

The number of bacteria present in human milk reported by other authors range between 1.5 and 4.0 log^10^ CFU/ml, with higher values in colostrum, between 3.14-4.22 log^10^ CFU/ml, which can increase to 6 log^10^ CFU/mL in cases of mastitis ^19^. In this study, we found a lower number of microorganisms in the samples, 2.73 log^10^ CFU/ml (range 1.30 to 3.47), a reduction associated with the fact that only microorganisms cultivable under the conditions described were included. There is no doubt that the application of molecular techniques based on the massive sequencing of r16S ribosomal subunit genes allows for the accurate assessment of the microbial diversity of human milk. However, culture-based techniques may be a viable alternative for studying the milk microbiota when sequencing techniques are not available. Tabit *et al*., in their review of molecular techniques in the study of milk, report that PCR-RFLP is one of the methodologies with the highest reproducibility of results, although it does not have much discriminatory power ^20^^).^

The main limitations of this study should be considered in order to interpret our results, these being mainly the small sample size, the lack of randomization in its selection, and the absence of information regarding the mother’s diet as a determining factor of the microbiota. The use of culture techniques in the study of the microbiota limits the findings to cultivable microorganisms. The usefulness of PCR-RFLP for identifying microbial isolates only allows for identification at the genus level.

In conclusion, the findings of our study constitute a starting point in understanding the microbial composition of human colostrum, considering the particular context and environmental conditions of the Colombian Caribbean. These results are consistent with reports from other parts of the world, which point to the dominance of genera such as *Staphylococcus, Lactobacillus*, and *Enterococcus*. It is important to implement mass sequencing techniques to consolidate this finding and achieve species- and subspecies-level identification of the microorganisms present in colostrum. The identification and characterization of potential probiotics among the isolated microorganisms is of great interest to medicine and industry.
