# Supplementary material for: Identification by PCR-FRLP of bacteria present in the colostrum of women residing in Barranquilla, Colombia
Source: Rev Peru Med Exp Salud Publica. 2025 Jun 11;42(2):184–9. doi: 10.17843/rpmesp.2025.422.14321 (PMC12377886; doi:10.17843/rpmesp.2025.422.14321)
Supplement: Translation

# Translation: Identificación mediante PCR-FRLP de bacterias presentes en el calostro de mujeres residentes en Barranquilla, Colombia

**Authors:** Luz A. Sarmiento-Rubiano, Leidys Goenaga, Marianella Suarez-Marenco, Clara Gutierrez-Castañeda, Carmen M. Sarmiento, Jimmy Becerra Enríquez

**Affiliations:** 1 Grupo de Investigación Alimentación y Comportamiento Humano, Universidad Metropolitana, Barranquilla, Colombia.; 2 Grupo de Investigación Gestión Ecológica y Agroindustrial, Programa de Microbiología, Universidad Libre, Barranquilla, Colombia.; 3 Medicina preventiva y comunitaria, Universidad del Sinú, Cartagena, Colombia.

**Keywords:** Calostro, Polimorfismo de Longitud del Fragmento de Restricción, Microbiota, Lactancia Materna

## Abstract

Con el objetivo de aislar e identificar bacterias presentes en el calostro de mujeres de la ciudad de Barranquilla, ubicada en el Caribe colombiano, se realizó un estudio descriptivo en 55 muestras de calostro, las cuales fueron cultivadas en agares M17, MRS y TOS, e incubados bajo condiciones de aerobiosis y anaerobiosis. Se obtuvieron 350 aislamientos de microorganismos, de los cuales 296 fueron identificados a nivel de genero mediante PCR-RFLP con los enzimas *Hae*III y *Rsa*I. Se utilizó el programa en línea *kodebio.shinyapps.io/RFLP-inator* para la identificación in *silico* de los aislamientos. Se identificaron siete géneros bacterianos hipotéticos: *Staphylococcus, Lactobacillus*, *Enterococcus, Bifidobacterium, Corynobacterium, Streptococcus y Leuconostoc, siendo* los dominantes: *Staphylococcus, Lactobacillus* y *Enterococcus*, los cuales representan el 61,1% de los microorganismos identificados. Los resultados de esta investigación constituyen un punto de partida en el conocimiento de la composición microbiana del calostro humano, considerando el contexto particular y las condiciones ambientales del Caribe colombiano.

## INTRODUCCIÓN

Actualmente se han identificado más de 590 géneros bacterianos en la leche humana, pertenecientes principalmente a los *phylum Firmicutes*, *Actinobacteria* y *Protebacteria*, siendo especialmente dominantes los géneros *Staphylococcus*, *Streptococcus*, *Lactobacillus*, *Pseudomonas*, *Bifidobacterium*, *Corynebacterium*, *Enterococcus*, *Acinetobacter*, *Clostridium* entre otros ^1^.

La leche humana tiene todos los componentes y nutrientes necesarios para el adecuado crecimiento, desarrollo y protección de los neonatos, y es su principal fuente de bacterias comensales, mutualistas y potencialmente probióticas ^(^^2^. Las bacterias aportadas por la leche materna cumplen diversas funciones entre las que se destacan: disminución en la incidencia y severidad de infecciones ^3^; maduración del sistema inmunológico ^4^; defensa contra bacterias patógenas por la presencia de fagos bacterianos ^5^; la exposición al microbioma lácteo materno favorece la salud intestinal del neonato, y su desarrollo físico y mental ^(^^6^.

La composición de la microbiota de la leche humana varía según el periodo de lactancia y factores como la higiene y alimentación de la madre, su microbiota intestinal, consumo de antibióticos, localización geográfica, estilos de vida y aspectos genéticos, epigenéticos y étnicos ^7^. El calostro es producido por las glándulas mamarias desde el momento del parto y durante los seis días posteriores a este, es abundante en citoquinas, péptidos antimicrobianos, anticuerpos, hormonas y diferentes compuestos bioactivos ^(^^2^. La microbiota del calostro está fuertemente asociada al ambiente materno, así como al tipo de parto (natural o cesárea), la evolución del calostro a leche madura tiene importantes cambios en su microbiota, en los que la interacción del incipiente microbioma oral del lactante con la glándula mamaria es fundamental ^8^.

Estudiar los microorganismos de la leche humana en diferentes condiciones ambientales y poblacionales contribuye al conocimiento de este fluido y su potencial impacto en la salud del lactante y la madre. Técnicas dependientes e independientes de cultivo permiten identificar una gran variedad de bacterias; cada una de estas técnicas con ventajas y desventajas que se evidencian en los resultados obtenidos. No obstante, las técnicas de cultivo permiten el aislamiento y conservación de microorganismos con potenciales características probióticas. El objetivo de este estudio fue aislar e identificar bacterias del calostro de mujeres de la ciudad de Barranquilla, en el Caribe colombiano, utilizando la técnica de cultivo y su posterior identificación mediante PCR-FRLP (Restriction Fragment Length Polymorphism).

**MENSAJE CLAVE**

Motivación para realizar el estudio. Existe poca información sobre la microbiota presente en el calostro de mujeres que habitan el Caribe colombiano. Identificar y aislar microorganismos en este fluido es de interés tanto para la medicina como para la industria.

Principales hallazgos. Los géneros bacterianos dominantes encontrados en el calostro fueron Staphylococcus, Lactobacillus y Enterococcus. Se aislaron microorganismos con potencial probiótico que deben ser estudiados con mayor detalle.

Implicancias en salud pública. Conocer la microbiota presente en el calostro permite comprender su contribución a la salud del lactante y desarrollar estrategias para potenciar el impacto beneficioso de estos microorganismos en su desarrollo.

## EL ESTUDIO

Se realizó un estudio descriptivo transversal, que incluyó a 55 mujeres posparto, atendidas en el servicio de maternidad del Hospital Metropolitano de la ciudad de Barranquilla (Caribe colombiano) y que cumplieron los siguientes criterios de inclusión: ser mayor de edad, haber tenido un mínimo 37 semanas de gestación, parto vaginal sin complicaciones ni consumo de antibióticos, presentar una condición saludable y haber accedido voluntariamente a participar mediante la donación de 3 a 5 ml de calostro, recolectados entre 12 a 48 horas posparto. El calostro se obtuvo tras el lavado de los pezones mamarios con jabón neutro y agua estéril, utilizando un extractor de leche eléctrico (Medela Swing), en condiciones estériles. Las primeras dos gotas de calostro fueron descartadas y 3 a 5 ml de muestra se recolectaron en recipiente esteril y fueron almacenadas a -80 ºC hasta su análisis.

Para aislar las bacterias del calostro, se realizó siembra directa de 100 µl de muestra en los agares de cultivo MRS (Man Rogosa Sharpe, para el aislamiento de bacterias ácido-lácticas), M17 (para *Streptococcus* lácticos) y agar Propionato TOS (para bifidobacterias). Los agares se incubaron a 37 °C durante 72 horas (MRS y M17 en condiciones de aerobiosis y anaerobiosis -sistema Anaerogen de Oxoid y TOS solo en anaerobiosis-). Las colonias aisladas desde los diferentes cultivos fueron identificadas morfológicamente mediante tinción de Gram, recultivadas en medio de cultivo liquido de acuerdo con el agar de procedencia o en Caldo Infusión Cerebro Corazón (BHI) y crio preservadas a -80 °C con glicerol al 20%, para futuros estudios.

La identificación a nivel de género de las bacterias aisladas del calostro se realizó mediante PCR-RFLP. Se amplificaron **≈**1508 pares de bases del r16S ADN, mediante PCR de colonia con los iniciadores universales 27F: 5’-AGAGTTTGATCCTGGCTCAG-3’ y 1492R: 5’-GGTTACCTTGTTACGACTT-3’ ^9^. Cada 50 µl de reacción de PCR contenía: tampón 1X (BIOTOOLS), 1.5 mM MgCl2, 200 μM de dNTPs, 20 pmol de cada cebador, 1U de Taq polimerasa (BIOTOOLS) y una pequeña porción de colonia adicionada con palillo estéril. *Lactococcus lactis* NZ900 fue utilizado como control positivo de la reacción. El programa de amplificación fue: cinco minutos a 96 °C; 35 ciclos: 95 °C por 30 segundos, 60 °C por 30 segundos, 72 °C un minuto; y un ciclo de 72 °C siete minutos (Termociclador BIO-RAD). Los productos de PCR se visualizaron en geles de agarosa al 0,8% coloreados con Syber-Green. La electroforesis se hizo con Buffer TAE 1X, a 80V durante 30 minutos (electroforesis horizontal de Bio-Rad).

Para RFLP, los amplificados por PCR fueron sometidos a digestión en reacciones separadas, con las enzimas *Hae*III (GC^GC) y RsaI (GT^AC) de acuerdo con las recomendaciones del fabricante (*New England Biolab*) (10 µl del producto de PCR, 2 unidades de enzima, 2 µl buffer 10X en volumen final de 20 µl) las reacciones se incubaron 3 horas a 37 °C. Los productos de digestión se sometieron a electroforesis en geles de agarosa al 2% con Syber-Green y se visualizó el perfil de bandas generado en un fotodocumentador Biorrad ChemiDoc™ XRS. Los tamaños de bandas obtenidas se compararon con el marcador de peso molecular para ADN HyperLadder™ 100pb DNA-Bioline.

Los perfiles de bandas resultantes fueron clasificados en ocho grupos de microorganismos que mostraron perfiles de restricción idénticos con los dos enzimas. Utilizando el programa en línea *kodebio.shinyapps.io/RFLP-inator*, que permite la identificación bacteriana a partir de simulación in *silico* de perfiles de restricción del r16S ADN, fue posible la identificación a nivel de genero de los aislamientos obtenidos. Se utilizo como control positivo *Lactococcus lactis* subsp. *cremoris* NZ9000 (secuencia GenBank NCBI NC_017949.1) cuyos perfiles de restricción con *Hae*III y *Rsa*I, fueron verificados *in silico*.

Se realizo un análisis estadístico descriptivo. El crecimiento microbiano se expresa como log^10^ UFC/ml. La identificación de los microorganismos en PCR-RFLP se muestran con frecuencias relativas.

El protocolo de investigación de este estudio fue aprobado por el Comité de Ética de la Universidad Metropolitana (código 0340517) y todas las participantes firmaron el consentimiento informado de acuerdo con la Resolución 8430 de 1993 del Ministerio de Salud y Protección Social de Colombia y la Declaración de Helsinki.

## HALLAZGOS

De las 55 muestras de calostro cultivadas, solo el 90,9% (n=50) mostro crecimiento microbiano en alguno de los medios de cultivo en las condiciones descritas. El número promedio de microorganismos aislados de cada calostro fue 2,73 log^10^ UFC/ml (rango 1,30 a 3,47). El crecimiento microbiano en cada uno de los medios de cultivo y condiciones evaluados tuvo un valor promedio de 2.39 log^10^ UFC/ml (tabla 1). Teniendo en cuenta las diferentes condiciones de cultivo y morfología diferencial de las colonias, fue posible el aislamiento de 350 microorganismos. Mediante tinción de Gram se determinó la morfología microscópica de los aislamientos; en el medio M17 se observaron principalmente cocos grampositivos, en MRS cocobacilos grampositivos y bacilos grampositivos, y en el medio TOS cocos y cocobacilos grampositivos.

**Tabla 1:** Número de microorganismos aislados a partir de muestras de calostro desde cada medio de cultivo y condiciones diferentes.

Medio de cultivo	Condición	Muestras con crecimiento n=50 (%)	Promedio log^10^ UFC/ml	Número de microorganismos aislados (n=350)
M17	Aeróbica	40 (80)	2,42	128
	Anaeróbica	28 (56)	2,27	72
MRS	Aeróbica	16 (32)	2,41	53
	Anaeróbica	8 (16)	2,59	27
TOS	Anaeróbica	23 (46)	2,36	70

UFC: unidades formadoras de colonia, M17: medio de cultivo Agar M17, MRS: medio Man Rogosa Sharpe, TOS: medio agar propionato.

De todos los microorganismos aislados, fue posible la amplificación por PCR (**≈**1508 pb) del r16S ADN. La digestión con las enzimas *HaeIII* y *RsaI*, dio lugar a 332 y 335 perfiles de bandas. Debido a que no todos los aislados mostraron un patrón definido (bandas difusas, imperceptibles o no digestión del fragmento de ADN), fueron seleccionados solamente 296 aislados para la identificación (figura 1). Dado que muchas de las bacterias presentaron el mismo perfil de bandas con las dos enzimas, se procedió a agrupar los perfiles obteniéndose ocho patrones de restricción de los 296 microorganismos (tabla 2).

**Figura 1:** Flujograma del proceso de cultivo e identificación de microorganismos en el calostro humano.

^a^ Muestra las causas de exclusión de microorganismos en el proceso.

**Tabla 2:** Identificación de los géneros bacterianos aislados a partir de las muestras de calostro mediante PCR-RFLP.

Patrón	Medios de cultivo	Tinción de Gram	Identificación perfil de bandas por RFLP- inator			% MI (n=296)
			^a^ ***Hae*III**	^a^ ***Rsa*I**	Microorganismos hipotéticos	
1	M17 aerobiosis M17 anaerobiosis	Cocos Gram +	1200, 310	630, 490, 380	*Hae*III: S004065075 *Staphylococcus aureus* 08BA02176 *Rsa*I: S000404448 uncultured rumen bacterium BE38	16,4
2	M17 aerobiosis M17 anaerobiosis	Cocos Gram +	1200, 310	500, 490, 410, 120	*Hae*III: S004443882 *Staphylococcus epidermidis* SEI *Rsa*I: S001095749 *Staphylococcus epidermidis* SSL08	20,2
3	M17 anaerobiosis M17 anaerobiosis	Cocos Gram +	600, 460, 290, 120, 30	900, 350, 150, 120	*Hae*III: S000892225 *Enterococcus mundtii* HDYM-22 *Rsa*I: S002949963 *Enterococcus* sp. PPC34A	12,4
4	M17 anaerobiosis	Cocos Gram +	580 450, 310, 120	620, 360, 260, 150, 120	*Hae*III: S000470127 *Streptococcus salivarius* subsp. null DL1 *Rsa*I: S002032101 *Streptococcus* sp.	8,2
5	MRS aerobiosis MRS anaerobiosis	Bacilos Gram +	570, 460, 330, 120, 30	900, 350, 140, 110	*Hae*III: S003260210 *Lactobacillus pentosus* ZU 28 *Rsa*I: S003260206 *Lactobacillus pentosu*s ZU 24	15,1
6	MRS anaerobiosis	Cocobacilos Gram +	1100, 270, 70, 45	490, 400, 120, 100	*Hae*III: S000430949 *Leuconostoc pseudomesenteroides* RO1 *Rsa*I: S000978801 *Leuconostoc pseudomesenteroides* QL28	7,7
7	TOS anaerobiosis	Bacilos Gram +	460, 360, 160, 95, 85, 65, 55, 35	410-210-160-150-115-85	*Hae*III: S001576581 *Corynebacterium* sp. ICIRC105 *Rsa*I: S000721364 *Corynebacterium aurimucosu*m AE1-3	8,5
8	TOS anaerobiosis	Bacilos Gram +	260, 200, 150, 100, 90, 70, 50, 30, 20	630, 360, 250, 150, 120	*Hae*III: S000871434 *Bifidobacterium animalis* subsp. lactis IDCC 4301 *Rsa*I: S000871434 *Bifidobacterium animalis* subsp. lactis IDCC 4301	11,5
Control	M17 anaerobiosis	Diplococos Gram +	457, 410, 309, 187, 123, 22	890, 355, 146, 83, 34	*Lactococcus lactis* subsp. cremoris NZ9000, (NC_017949.1)	

Los perfiles de bandas obtenidos con cada enzima de digestión se muestran de acuerdo con los tamaños de bandas generados.

a HaeIII y RsaI son enzimas de restricción utilizados en la digestión.

MI: microorganismos identificados. RFLP: Restriction Fragment Length Polymorphism, M17: medio de cultivo Agar M17, MRS: medio Man Rogosa Sharpe, TOS: medio agar propionato.

La identificación de los géneros bacterianos se realizó teniendo en cuenta su crecimiento en el medio de cultivo, la morfología en la tinción de Gram, y los resultados del análisis del patrón de bandas *in silico* utilizando el programa *kodebio.shinyapps.io/RFLP-inator*.

Del medio de cultivo M17 se logró el aislamiento e identificación del mayor número de microorganismos 57,2% (n=200). Con crecimiento en condiciones de aerobiosis y anaerobiosis, se identificó bacterias del género *Staphylococcus* (patrones 1 y 2) que mostraron un perfil de bandas común a la digestión con *Hae*III, pero diferente con *Rsa*I; y bacterias del género *Enterococcus* (patrón 3). Los microorganismos crecidos en agar M17 solo en condiciones anaerobias, fueron el 8,2% de los aislamientos, que se identificaron como bacterias del género *Streptococcus* (patrón 4).

En el medio MRS se aisló el 22,8 % de los microorganismos identificados, con crecimiento en condiciones aerobias y anaerobias, se identificaron *Lactobacillus* (patrón 5) y de crecimiento solo en anaerobiosis *Leuconostoc* (patrón 6). Las bacterias recuperadas e identificadas en el medio TOS bajo la condición anaeróbica (patrones de bandas 7 y 8) que representan el 20% de los aislamientos, pertenecen a los géneros *Corynebacterium* sp. y *Bifidobacterium* respectivamente (tabla 2).

## DISCUSIÓN

En este estudio, se aislaron e identificaron mediante PCR-RFLP, siete hipotéticos géneros de bacterias presentes en 50 muestras de calostro humano, pertenecientes a los géneros *Staphylococcus, Lactobacillus*, *Enterococcus, Bifidobacterium, Corynobacterium, Streptococcus y Leuconostoc, siendo* los géneros dominantes *Staphylococcus, Lactobacillus* y *Enterococcus* que corresponde al 61,1% de los 296 microorganismos identificados, de 350 aislamientos.

Otros autores utilizando métodos dependientes de cultivo, han identificado en leche humana, como géneros bacterianos dominantes, a *Staphylococcus, Streptococcus, Corynobacterium* y *Cutibacterium,* y en menores proporciones *Lactococcus, Enterococcus, Lactobacillus, Leuconostoc, Weisella,* y *Bifidobacterium*^2^^,^^10^. Al igual que en este estudio, una flora láctea dominante de *Staphylococcus* ha sido reportada previamente. Por ejemplo, en mujeres de áreas urbanas de la China, mediante secuenciación del r16S ADN se observó que más del 40% del microbiota lácteo correspondía a bacterias de los géneros *Staphylococcus* y *Streptococcus*^11^. Un metaanálisis que incluyó doce estudios de diferentes lugares del mundo encontró que los géneros dominantes en la leche, indiferente de la ubicación geográfica son los *Staphylococcus* y *Streptococcus*^12^*.* Al respecto hay que destacar que la microbiota evoluciona desde el calostro a la leche madura y que estudios como el de Jiménez *et al.* en 2008, en calostro de 36 mujeres españolas, encontró como géneros dominantes a los *Staphylococcus* y *Enterococcus,* y en menor proporción *Lactobacillus*^13^*.* Resultados coherentes con los encontrados en este estudio donde los *Enterococcus* y *Lactobacillus* fueron los géneros más frecuentes después de *Staphylococcus*.

La dieta de la madre, la diabetes, la obesidad e incluso el sexo del bebe, entre otros, son factores identificados como influyentes en la composición de la microbiota del calostro ^(^^14^. Xie *et al*., evaluando 97 madres sanas de la isla china de Hainan, encontraron que el origen étnico de los individuos puede ser un factor importante asociado a la diversidad del microbioma del calostro ^(^^15^. En el caso colombiano y de manera específica en el Caribe, existe una gran diversidad étnica y cultural caracterizada por la mezcla de ascendencia africana, europea y nativa americana ^16^. Esta diversidad da origen a perfiles étnicos y genéticos tan variados que resultaría muy difícil establecer relaciones entre la microbiota láctea y la etnia, más aún cuando solo se cuenta con una identificación a nivel de género de las especies presentes en el calostro. Respecto a la dieta materna, se conoce que el consumo de macronutrientes, principalmente grasa, modifica la calidad nutricional del calostro ^17^. La disponibilidad de nutrientes tanto en el fluido lácteo como en la mucosa intestinal del niño afecta en variedad y cantidad de las poblaciones bacterianas existentes, en este estudio la dieta materna no fue evaluada. En Colombia existen pocos estudios sobre la microbiota láctea humana, entre ellos, Londoño-Sierra *et al.*, evaluando 101 mujeres lactantes de la ciudad de Medellín, demostraron una relación directa entre el estado nutricional y dieta materna, con los géneros microbianos dominantes en la leche ^(^^18^^)^ .

El número de bacterias presentes en la leche humana reportado por otros autores podría oscilar entre 1,5 y 4,0 log^10^ UFC/ml, con valores mayores en el calostro, entre 3,14-4,22 log^10^ UFC/ml, que pueden aumentar hasta 6 log^10^UFC/mL en casos de mastitis ^19^. En este trabajo se observó un número menor de microorganismos en las muestras, 2,73 log^10^ UFC/ml, (rango 1,30 a 3,47), reducción que está asociada a que solo se incluyeron microorganismos cultivables en las condiciones descritas. Es indudable que la aplicación de técnicas moleculares basadas en la secuenciación masiva de los genes de la subunidad ribosomal r16S ADN permiten evaluar la diversidad microbiana de la leche humana de manera muy acertada. Sin embargo, las técnicas basadas en cultivo pueden ser una alternativa viable para el estudio de la microbiota láctea cuando las técnicas de secuenciación no están disponibles. Tabit *et al*., en su revisión sobre las técnicas moleculares en el estudio de la leche, refieren que la PCR-RFLP es una de las metodologías con mayor reproductividad en los resultados, aunque no con mucho poder discriminatorio ^20^.

Las principales limitaciones de este estudio deben ser consideradas para mejorar la interpretación de los resultados, siendo estas principalmente el pequeño tamaño de muestra, la falta de aleatoriedad para su selección, y la ausencia de información en relación con la alimentación de la madre como factor determinante del microbiota. El uso de técnicas de cultivo en el estudio de la microbiota limita los hallazgos a microorganismos cultivables. La utilidad de PCR-RFLP para la identificación de aislamientos microbianos solo permite la identidad a nivel de género.

En conclusión, los hallazgos de este estudio constituyen un punto de partida en el conocimiento de la composición microbiana del calostro humano, considerando el contexto particular y las condiciones ambientales del Caribe colombiano. Estos resultados son coherentes con los reportes de otras partes del mundo, que señalan la dominancia de géneros como *Staphylococcus*, *Lactobacillus* y *Enterococcus.* Es importante implementar técnicas de secuenciación masiva que permitan consolidar este hallazgo y lograr la identificación a nivel de especie y subespecie de los microorganismos presentes en el calostro. La identificación y caracterización de potenciales probióticos entre los microorganismos aislados resulta de gran interés para la medicina y la industria.
